# How COVID-19 has changed the utilization of different health care services in Poland

**DOI:** 10.1186/s12913-024-10554-7

**Published:** 2024-01-18

**Authors:** Magdalena Mrożek-Gąsiorowska, Marzena Tambor

**Affiliations:** Department of Health Economics and Social Security, Institute of Public Health, Faculty of Health Sciences, Jagiellonian University Medical College, 8 Skawinska Street, 31-066 Krakow, Poland

**Keywords:** Health care utilization, COVID-19, Public health, Prevention, Rehabilitation, Poland

## Abstract

**Background:**

The COVID-19 pandemic has affected health care systems in many ways, including access to and the use of non-COVID services. The aim of the study was to assess the impact of the pandemic on the utilization of different public health care services in Poland.

**Methods:**

The aggregated data on health care users and provided services for the years 2015/2016–2021 were used to analyse the changes in health care utilization during the pandemic and deviations from pre-pandemic utilization trends. Quantitative analysis was complemented with qualitative descriptions of the changes in principles of health care provision during the pandemic.

**Results:**

The results show a considerable drop in the provision of most health care services in 2020 that in some cases disturbed pre-pandemic utilization trends and was not made up for in 2021. The most significant decrease has been observed in the field of preventive and public health services, as well as rehabilitation. The provision of these services was put on hold during the pandemic.

**Conclusions:**

The accumulated COVID-19-related “health debt” urgently calls for government actions to strengthen disease prevention and health promotion in Poland.

**Supplementary Information:**

The online version contains supplementary material available at 10.1186/s12913-024-10554-7.

## Background

The COVID-19 pandemic has affected all sectors and industries around the world. Among the most disrupted was the health care sector, which had to deal with the treatment and prevention of the new disease. The urgent need to allocate available resources to fight the pandemic limited their availability for non-COVID services. This, coupled with patients forgoing the use of health care services due to fear of being exposed to coronavirus, has resulted in the reduction of non-COVID-related health care utilization.

Extensive scientific evidence confirms the detrimental impact of the COVID-19 pandemic on health care use [1]. This evidence highlights the effects of the pandemic on different type of health services (e.g. prevention and screening programmes [2], mental health services [3–5], emergency health care [6], blood transfusion services [7], services for sexual and reproductive health [8], diagnostic tests or specific therapy, such as radiology [9]), or on specific patient groups and health problems (e.g. oncological patients [10–12], patients with cardiovascular diseases [13–15], chronic kidney disease [16], and infectious diseases other than COVID-19 [17–19]). There are also publications that look more broadly at the use of health care in a particular country (e.g. India [20]) and region (e.g. South-East Asia [21]).

In Poland, one of the largest EU countries, the health care system was also severely affected by the COVID-19 pandemic. The first cases of the disease were recorded in this country in March 2020 and the state of epidemic was in force till May 15, 2022 [22]. During the different waves (first wave– spring 2020, second wave– autumn 2020, third wave– spring 2021, fourth wave– autumn 2021 and fifth wave– winter/spring 2022), several health care facilities were completely closed or the provision of health services was severely limited in line with the government’s strategy and recommendations [23–25]. To ensure access to health care, some services were partly provided in the form of e-visits, particularly primary care. At the same time, output-based financing was put on hold to secure health care providers’ income [26].

Despite the sizeable published evidence, there is no comprehensive analysis of the use of health care services in Poland during the COVID-19 pandemic using aggregated country-level data. The previous publications on Poland either focus on selected types of health services or patients with particular diseases [27–39]. Among the Polish studies, there are also one-centre or multicentre studies on specific types of health services [40–47], or surveys carried out among specialists or patients, which assess the impact of the pandemic on particular areas of the health care system [48–54].

Thus, in this study, we aim to analyse the use of different publicly financed health care services in Poland in 2020–2021 in comparison with the utilization in the pre-pandemic years of 2015–2019. We complement the results of the quantitative analysis with qualitative descriptions of the conditions in which specific services were provided during the pandemic. Our analysis covers a broad range of services, i.e. primary care, outpatient specialist care, hospital care, prevention, dental care, psychiatric care, rehabilitation, long-term care, and palliative care, as well as medicines and medical devices. This allows us to draw conclusions on the effects of the pandemic on different areas of health care and discuss the factors that might have contributed to the observed variation. By analysing the years of 2020 and 2021, we can also ascertain whether certain areas of health care adjusted faster than others to the new conditions and were able to resume the provision of services. The recommendations that result from our research might help to improve health care systems’ responses and resilience to future external shocks such as pandemics.

## Methods

This study combines qualitative analysis on the conditions for provision of health care services during the COVID-19 pandemic, and quantitative analysis of aggregated country-level data on health care utilization within the statutory health care system in Poland.

The qualitative analysis includes data on the continuity of health care provision during the pandemic, modes of health care provision, and the methods used by the National Health Fund (NHF), the main payer institution, to pay for services. The analysis is largely based on a review of legislation issued in 2020 and 2021 by the public authorities, mainly the Council of Ministers, the Minister of Health, and the President of the NHF. The regulations were hand searched through two dedicated databases:

- The Internet System of Legal Acts (http://isap.sejm.gov.pl), which includes consolidated texts of normative legally binding acts published in the Journal of Laws of the Republic of Poland (Dziennik Ustaw) and the Official Gazette of the Republic of Poland “Monitor Polski”.
- The database of the acts of the NHF (https://baw.nfz.gov.pl/NFZ/tabBrowser/mainPage).

The quantitative analysis was based on publicly available data provided by the NHF. The NHF publishes annual reports that include information on the number of health care users and the volume of specific services provided within each scope (primary health care, outpatient specialist care, hospital care, etc.) [55]. The reports concern health care services financed from statutory insurance contributions, and do not cover health benefits financed from the state budget or by territorial self-governments, which constitute a minor part of the Polish health care system (i.e. government transfers account for 11% of current health expenditure and 15% of public current health expenditure [56]). However, during the pandemic period, the NHF received additional funds to finance services for COVID patients, and these services are covered by the reports.

For this study, we extracted data from seven annual reports for 2015–2021. Nevertheless, due to the changes in reporting method introduced in 2016, comparable data for 2015 were not available for some types of services, thus, we used data for the years 2016–2021 for these services. The following indicators were analysed:

- the number of users, i.e. people who used any health care services within a given scope at least once during the year (e.g. primary health care).
- the number of specific health care services provided during the year (e.g. the number of visits, completed inpatient stays, or certain types of services) and the number of people using specific services within a given scope (e.g. children who underwent screening tests via primary care).

We have examined the changes in these indicators during two pandemic years (2020 and 2021). Based on available data for pre-pandemic years, we also performed a linear trend analysis, using the least-squares method, forecasting the trend for 2020–2021. Then, we compared the forecasted values with the data available for the two pandemic years to see how the actual utilization during in 2020 and 2021 deviated from the trend. Data were extracted in December 2022 and analysed using MS Excel.

The analysis covers the following types of services:

- Primary health care, including treatment and preventive services provided by a physician, nurse or midwife in an outpatient or home setting during working hours or nights and holidays, as well as services provided by a nurse or hygienist at school. We present data on consultations (face-to-face and teleconsultations) and, separately, on the numbers regarding preventive services for children (patronage visits, periodic health checks for children with screening tests) and adults (prevention of cardiovascular diseases), as well as school-based prevention. All primary health care services are provided free of charge to patients.
- Outpatient specialist care, i.e. services provided by specialists in outpatient clinics, which require a referral, except for psychiatric, oncological, gynaecological, and venereological care. We present data on consultations (conservative or interventional) and on the separately financed cost-intensive outpatient diagnostic services (nuclear medicine diagnostics, gastroscopy, colonoscopy, computed tomography, magnetic resonance imaging). In Poland, there is no patient cost-sharing in outpatient specialist care, however, long waiting times for services have been a significant problem.
- Hospital care, i.e. curative services provided in an inpatient setting (including one-day procedures), which require a referral except for emergencies. The analysis includes data on the total number of hospitalized people, and separately on patients hospitalized due to participation in a drug programme (innovative medicines) or chemotherapy. We also present data on the volume of selected inpatient services, i.e. arthroplasty and cataract surgery, for which data are separately provided by the NHF.
- Preventive health programmes, i.e. separately financed programmes delivered by providers selected in a separate tender competition, which are targeted at:
  - tobacco-related diseases, including chronic obstructive pulmonary disease (anti-tobacco counselling, education, spirometry test, anti-smoking therapy),
  - cervical cancer in women aged 25 to 59 (cytological examination, colposcopic and histopathological examination),
  - breast cancer in women aged 50 to 69 (mammography screening, breast ultrasound, biopsy).
  The analysis includes data on the number of services provided at the first stage of diagnosis and prevention, without in-depth examinations or further ordered services.
  Dental care, which covers a narrow scope of basic services. The cost of these services is fully financed by the NHF, and no obligatory patient cost-sharing or extra billing applies to dental care in the public system. Due to the low profitability of publicly financed services, only some providers offer public services, while most dentists provide only private services.
- Psychiatric care and addiction treatment, including services provided in inpatient, day, or outpatient settings.
- Medical rehabilitation, covering services provided in inpatient, day, outpatient, or home settings. These services are provided free of charge for patients based on a referral, but there is an upper limit of services to which patients are entitled.
- Health resort rehabilitation, i.e. services provided in health resorts in spa towns with the use of natural medicinal raw materials. The services are provided in a stationary setting (sanatorium or spa hospital), less often in outpatient settings, based on a referral which needs to be confirmed by the NHF. In the case of rehabilitation at a sanatorium, which is the most common form, the patient is required to co-pay for accommodation and food.
- Long-term care, i.e. services for dependent people, provided in stationary settings or at home by a long-term care nurse or a medical team in the case of mechanically ventilated patients. In stationary settings, the patient covers the costs of food and accommodation up to 70% of the patient’s monthly income.
- Palliative care, i.e. end of life care provided in stationary, home, or outpatient settings. The scope of these services also includes perinatal palliative care.
- Outpatient medicines, including prescribed medicines as well as foodstuffs for particular nutritional needs and simple medical products (dressings and blood glucose diagnostic strips). Access to outpatient medicines, with some exemptions, requires obligatory flat (PLN 3.20) or percentage patient co-payments (30% or 50%), and the fee above the reference price.
- Medical devices, e.g. limb prostheses, orthoses, crutches, wheelchairs, hearing aids, wigs, catheters, diaper pants and anti-bedsore mattresses. They are reimbursed differently than medicines and provided for patients based on an order issued by a physician, and approval by the NHF. For some medical devices, percentage co-payment is applied. Patients might be faced with an additional payment when choosing a product with a price higher than the reimbursement limit [57–59].

Based on the data published by the NHF, it is not possible to separate the services given to patients with COVID-19 from non-COVID-19 services. Thus, the presented data also include services for patients with COVID-19 or after recovering from COVID-19.

## Results

### Conditions for provision of health care services during the COVID-19 pandemic

The COVID-19 pandemic led to changes in the principles of health care provision. Various activities focused on securing additional services for people infected with coronavirus, detecting new cases of infection, and vaccination against COVID-19, which limited resources (personal, facilities, materials) for non-COVID-19 patients. An increased sanitary regime was introduced, which resulted in, among other things, longer consultation times, or a smaller number of people visiting clinics at the same time. Many restrictions were also introduced in the country (e.g. related to mobility), and some patients, for fear of coronavirus infection, cancelled scheduled consultations or hospitalizations. To reduce the risk of infection, teleconsultations were implemented, while provision on other services was temporarily put on hold. A package of instruments to strengthen the financial stability of health care providers was developed shortly after the start of the pandemic [60]. For providers paid using activity-based methods, an advance payment for contracted services was introduced. Hence, providers could receive payments regardless of their activities. Payments, at the request of providers, could also be made faster and more frequently [60–62]. All health care institutions that provided services within the publicly financed system received add on payments of 3% of each bill to cover the cost of the elevated sanitary regime [60, 63, 64]. Nevertheless, the shortage of personal protection equipment was a common problem at the beginning of the pandemic. More details on each type of service are presented below and in Table 1.

#### Primary health care

During the COVID-19 pandemic, capitation method continued to be the main mechanism to finance primary care, along with a fee-for-service method used to pay for specific services and lump sum payments to finance night and holiday care.

In response to the pandemic, in March 2020, e-visits, most often by telephone (teleconsultation), were introduced [23, 65]. Over time, in some primary health care facilities, teleconsultations became the dominant form of contact with physicians, comprising even 90% of all consultations [66], which raised concerns regarding accessibility and the quality of primary care. For this reason, in March 2021, regulations were implemented to oversee the provision of e-consultations (obligatory reporting) and limit their number in favour of traditional visits (an obligation for traditional consultation instead of an e-visit in certain cases) [67]. In June 2021, additional financial mechanisms were introduced to motivate primary care providers to reduce the share of teleconsultations in the total number of consultations through a higher capitation rate in the case of a low e-visit rate [66].

#### Outpatient specialist care

Before the pandemic, two main methods were used to pay for outpatient specialist care, i.e. a global budget (for hospital clinics within the so-called ‘hospital network’) and fees per visit, adjusted for the number and types of services provided during a visit (for providers operating outside the hospital network). In addition, specific costly services were financed by fee-for-service. After the start of the pandemic, global budgets continued to be used until July 2021. On the other hand, providers paid on a per visit basis could be still paid based on their activity or they could receive an advance payment for services contracted in 2020 (in monthly instalments), and then make up for the lower provision in the next year (this deadline was eventually extended till the end of 2023) [68]. This allowed them to ensure their income when provision of services was lower. Additionally, in July 2021 the upper limits of services paid for by the NHF were lifted, which allowed providers to deliver more visits and be paid for them. Along with withdrawing the limits, per visit payment was applied to all outpatient specialist services, including the ones previously paid for by the global budget.

Similarly to primary care, e-visits were introduced in March 2020; however, they could be carried out only for patients continuing their care, depending on patient’s clinical condition but not for the first visit to a specialist [69].

#### Inpatient hospital care

Most hospital services are financed through the global budget (within the hospital network) or Diagnosis-Related Group (DRG) (outside the hospital network). There is also a broad range of services that are paid for with the fee-for-service method. As with other types of care paid for based on activity, after the outbreak of the pandemic, providers offering services not paid for by the global budget could receive an advance payment for contracted services. However, in 2021 not all hospitals were able to make up for services not provided in 2020, and they needed to utilize the extended deadline till 2023. The provision of non-COVID-19 services was especially restricted for hospitals which in 2020 (until April 1, 2022) were dedicated specifically for COVID-19 patients or those with COVID-19 wards.

#### Preventive health programmes

The services of preventive health programmes before and during the pandemic were paid for using the fee-for-service method. However, due to the state of the COVID-19 epidemic, from March to June 2020, the provision of prevention programme services was put on hold [23]. Providers had the possibility to request advance payments in instalments. To resume provision of services, in May 2020 the rules for safe conduct of examinations, including, e.g. health checks in children, were defined [70].

#### Dental care

The small range of dental services that are available for patients under public system is financed through the fee-for-service method. This method was also used to pay for dental services during the COVID-19 pandemic. Throughout the pandemic, dental health care facilities were opened. However, the sanitary regime was also increased, which together with patients’ fear of SARS-CoV-2 infection, might have limited the use of dental services. From March till September of 2020, the NHF financed dental services for people suffering from COVID-19 in dental buses, but only in emergency cases. For persons suspected of coronavirus infection, emergency assistance was provided only by selected dental clinics [23, 57].

#### Psychiatric care and addiction treatment

Most services in inpatient and day care are financed on a person-day basis while outpatient care is paid for with the fee-for-service method. These activity-based methods were also used to pay for services during the pandemic. However, providers could receive advanced payments for contracted services. During the pandemic, there was a significant reduction in the availability of psychiatric care and addiction treatment services as a result of the Ministry of Health recommendation to put on hold the provision of group therapies and services in day wards, particularly for children, youth, and seniors. The possibility of a televisit with an outpatient psychiatrist was introduced in March 2020 [60, 71].

#### Medical rehabilitation and health resort rehabilitation

As with psychiatric care, medical rehabilitation in stationary and day care is paid per day whereas outpatient and home rehabilitation is paid per service. Health resort rehabilitation (stationary and outpatient) is financed on a person-day basis. These methods were also used during the pandemic with the possibility of receiving advance payments for contracted services. Rehabilitation centres were temporarily closed from March 14 to June 14, 2020, and from October 24 to December 31, 2020, while health resort rehabilitation was unavailable even longer [23]. The possibility of providing home rehabilitation services using information and communication technology (ICT) systems was introduced; however, it was used to a small extent given the specificity of rehabilitation services. To increase financial resources for rehabilitation and thus, improve access to these services, in 2020, a programme entitled “Medical services of the National Health Fund for the disabled for 2020–2021” was launched [72]. Moreover, in April 2021 post-COVID rehabilitation services were introduced, which had been preceded by a pilot programme in July 2020 [73].

#### Long-term care and palliative care

During the pandemic, there were no changes in the methods used to pay for long-term care and end-of-life care. A per-day payment was used to pay for long term care and palliative care in home and stationary settings, and fee-for-service was used for outpatient palliative care. However, as with other types of services, providers could ask for advance payments. The facilities were not closed during the COVID-19 pandemic, but the possibility of visits and contact of relatives with a dependent person in residential care facilities was limited. There was also a shortage of medical staff and personal protective equipment in these facilities, particularly in the first year of the pandemic, on a much larger scale than in the case of other types of services [74].

#### Outpatient medicines and medical devices

The principles and methods of financing outpatient medicines and medical devices were not changed due to the COVID-19 pandemic. However, the beginning of the COVID-19 pandemic coincides with the introduction of e-prescriptions (January 2020) for medicines and medical products, which replaced paper prescriptions. This has enabled medical professionals to issue prescriptions during e-visits.

**Table 1:** Provider payment methods used to pay for health care services in Poland

Types of health services	Pre-COVID-19 pandemic provider payment methods	Changes to payment methods for non-COVID-19 services during the pandemic (2020–2021)
Primary health care	- capitation (physician, nurse, midwife, and school nurse or hygienist care including most of the preventive services provided under primary health care) - fee-for-service (selected expensive diagnostic tests and certain types of consultations including cardiovascular disease prevention services, cervical cancer prevention programme services, and midwife visits) - lump sum (night and holiday care)	- capitation payment also covering teleconsultations (2020) - financial incentives (an increased capitation rate) to reduce the share of teleconsultations (2021) - advance payment for contracted services paid on a fee-for-service basis (2020) - add on payments (3% of the bill) to cover the cost of the elevated sanitary regime
Outpatient specialist care	- per visit payment adjusted for number and type of services provided during a visit (services provided outside the hospital network) or global budget (services provided within the hospital network) - fee-for-service for services billed separately (e.g. cost-intensive diagnostic services and other groups of specialist services)	- possibility of visits in the form of teleconsultations (2020) - advance payment for contracted services paid on per visit basis and with fee-for service (2020) - lifting limits on services paid for per visit (July 2021) - applying per visit payment to all providers, including hospitals in the network (July 2021) - an add on payments (3% of the bill) to cover the cost of an elevated sanitary regime
Hospital care	- global budget (lump sum) (within the hospital network) or DRG (outside the network) - fee-for-service (specific or highly specialized services, e.g. diagnostics for organ transplantation and organ transplantation, electrochemotherapy, and radiotherapy services)	- advance payment for contracted services paid for with DRG and fee-for service (2020) - an increase in the prices of services by about 5% (2020) - add on payments (3% of the bill) to cover the cost of the elevated sanitary regime
Preventive health programmes	- fee-for-service	- an advance payment for contracted services (2020) - add on payments (3% of the bill) to cover the cost of an elevated sanitary regime
Dental care	- fee-for-service	- an advance payment for contracted services (2020) - add on payments (3% of the bill) to cover the cost of the elevated sanitary regime
Psychiatric care and addiction treatment	- person-day (most services in stationary and day care) or fee-for-service (outpatient care) - monthly lump sum (only centre for environmental psychological and psychotherapeutic care for children and adolescents)	- an advance payment for contracted services paid for on a per visit and fee-for service basis (2020) - possibility of a visit in a form of teleconsultations (2020) - add on payments (3% of the bill) to cover the cost of the elevated sanitary regime
Medical rehabilitation	- fee-for-service (outpatient and home settings) or person-day (stationary and day settings)	- an advance payment for contracted services (2020) - introduction of teleconsultation and new fee for teleconsultation (2020) - add on payments (3% of the bill) to cover the cost of the elevated sanitary regime
Health resort rehabilitation	- person-day (outpatient and stationary settings)	- an advance payment for contracted services (2020) - add on payments (3% of the bill) to cover the cost of the elevated sanitary regime
Long-term care	- person-day (home and stationary settings)	- an advance payment for contracted services (2020) - add on payments (3% of the bill) to cover the cost of the elevated sanitary regime
Palliative care	- fee-for-service (outpatient settings) or person-day (home and stationary settings)	- an advance payment for contracted services (2020) - add on payments (3% of the bill) to cover the cost of an elevated sanitary regime
Outpatient medicines	- based on the established financing limit per medicine package with total reimbursement budget for medicines of no more than 17% of the total NHF budget for health care	- no changes
Medical devices	- based on the established financing limit for a given medical device	- no changes

### Health care users

Table 2 presents data on the number of health care users for specific scopes of health care services, (i.e. patients who used any health care services within a given scope at least once a year) during two years of the pandemic (2020 and 2021) as well as pre-pandemic years (2015/2016–2019). The graphs presenting these data, together with trends estimated based on pre-pandemic utilization data are included in Appendix 1 Figure A.

In the pre-pandemic period, the number of patients using services was relatively stable, and for many services we did not observe statistically significant trends (*p* > 0.05) (see Appendix 1 Figure A). The exemption is the growing number of patients under drug programmes as a result of a higher number of innovative drug therapies available in Poland. We also observed a significant upward trend for palliative care, in particular home hospices and medical devices. On the other hand, the numbers of dental care users and outpatient specialist care users were decreasing.

For most of the analysed types of health care services, a large reduction in the number of health care users was observed in the first year of pandemic (2020) (Table 2). The largest percentage decrease between 2019 and 2020 was recorded for health resort rehabilitation (49.7%) and preventive health programmes (32.0%), while the least significant drop was observed for long-term care (1.8%), and psychiatric care and addiction treatment (5.8%). In 2021, the numbers of users increased for all scopes of services. Nevertheless, in most cases the increase was not large enough to return to pre-pandemic use. Particularly, the numbers of hospital care users and health resort rehabilitation users were much below the 2019 level.

The pandemic does not seem to have impacted the implementation of hospital drug programmes as the number of users continued to increase during the pandemic being only slightly below the projected values based on pre-pandemic utilization (see Appendix 1 Figure A). In the case of chemotherapy programmes, there was a small decrease in the number of users in 2020, which was, however, made up for in 2021. Likewise, the number of people receiving medical devices decreased between 2019 and 2020 by 8%, but then it increased in 2021 above the pre-pandemic level although slightly below the expected value. The volume of reimbursed outpatient medicines did not change significantly during the coronavirus pandemic.

**Table 2:** Health care users

Types of health services		N (thousands)								*p*-value^&^		% change		
		2015	2016	2017	2018	2019	2020	2021		2015/2016–2019		2020/ 2019	2021/ 2020	2021/ 2019
Primary health care		n/d	27793.6	28039.5	27931.1	27923.4	25885.2	27354.0		NS		-7.3%	5.7%	-2.0%
Outpatient specialist care		n/d	18126.5	17893.4	17337.0	17167.8	15189.3	15982.6		0.022		-11.5%	5.2	-6.9%
Hospital care	- inpatient services	n/d	9023.1	9158.8	9379.0	9251.1	7041.1	7589.1		NS		-23.9%	7.8	-18.0%
	- drug programmes	n/d	102.6	120.6	136.0	153.3	161.1	175.0		< 0.001		5.1%	8.6%	14.1%
	- chemotherapy	n/d	132.4	132.9	134.7	138.4	134.9	142.9		NS		-2.6%	5.9%	3.2%
Preventive health programmes		n/d	1657.3	1694.9	1590.0	1573.2	1070.0	1480.0		NS		-32.0%	38.3%	-5.9%
Dental care		n/d	7008.4	6785.1	6554.5	6495.1	4988.7	5447.2		0.025		-23.2%	9.2	-16.1%
Psychiatric care and addiction treatment		n/d	1684.1	1676.8	1664.5	1602.9	1510.5	1602.1		NS		-5.8%	6.1%	0.0%
Medical rehabilitation		n/d	3381.7	3357.1	3353.1	3270.4	2716.7	2956.1		NS		-16.9%	8.8	-9.6%
Health resort rehabilitation		n/d	407.9	401.7	404.1	406.1	204.3	265.6		NS		-49.7%	30.0	-34.6%
Long-term care		n/d	104.8	110.2	109.6	109.2	107.3	112.8		NS		-1.8%	5.1%	3.2%
Palliative care		n/d	88.5	91.9	95.2	101.2	90.9	93.1		0.012		-10.1%	2.4	-7.9%
Outpatient medicines*		251891.4	264579.0	257035.8	274241.9	255293.2	247015.7	248889.5		NS		-3.2%	0.8	-2.5%
Medical devices*		n/d	1617.7	1707.1	1758.9	1822.7	1671.0	1897.0		< 0.001		-8.3%	13.5%	4.1%

n/d– no data

^&^– *p*-value for linear trend

NS– Not Significant, p>0.05

^*^– Number of prescriptions

### Provided services and users of specific health care services

#### Primary health care

**Fig. 1:**
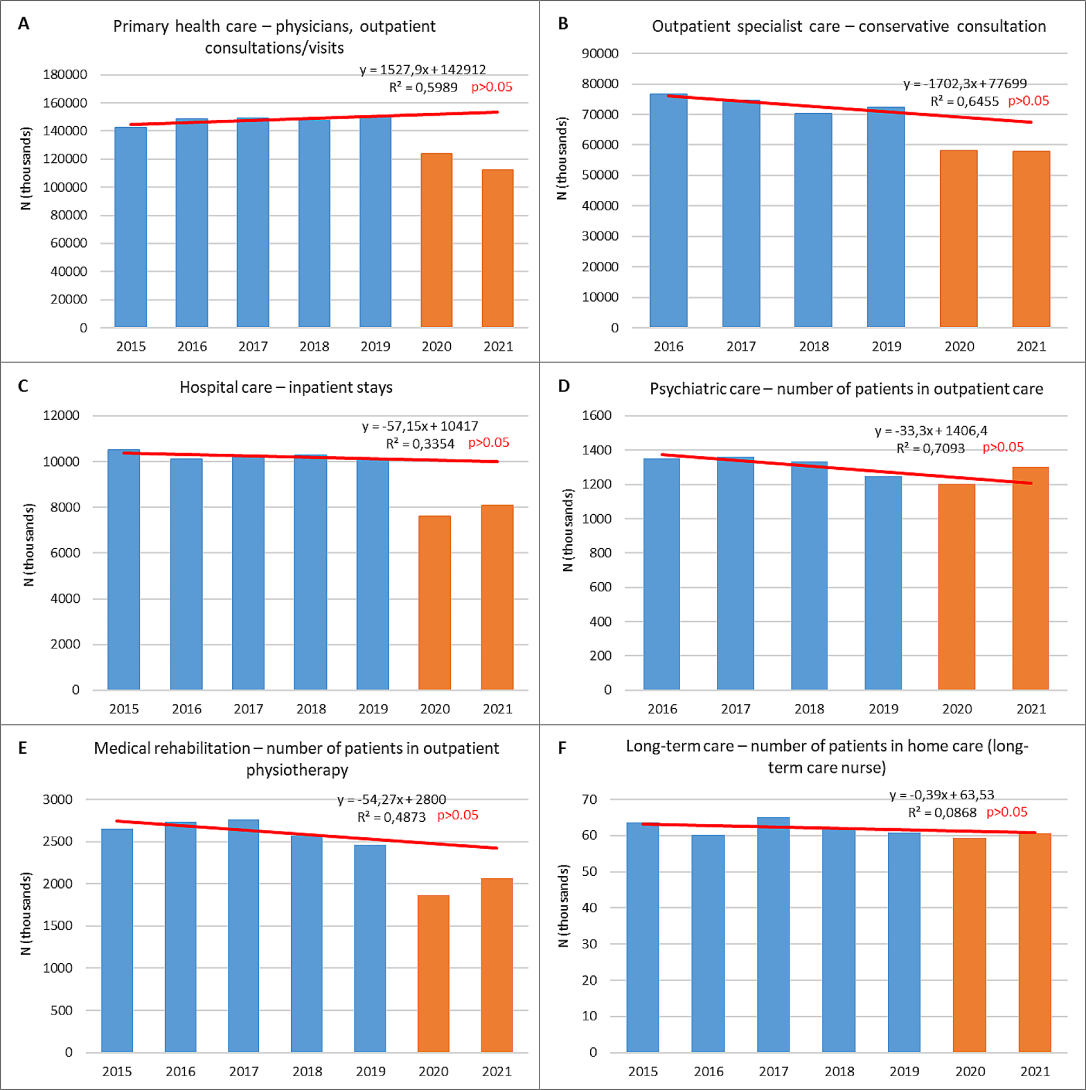
The utilization of most frequent health care services within a given scope in 2015/2016–2021. Blue bars– pre-pandemic years (2015/2016–2019). Orange bars– pandemic years (2020–2021). Red line– linear trend based on pre-pandemic data

In 2020, the total number of primary care visits decreased more significantly than the number of patients using primary health care services (Table 3). The smallest decreases were observed for outpatient midwife services and night and holiday care provided in home settings. Moreover, in the case of the night and holiday care, the decline was consistent with a statistically significant pre-pandemic downward trend. The number of the most frequently provided primary health care consultations, i.e. physician outpatient consultations (face-to-face or teleconsultations), was increasing in the pre-pandemic period, though the linear trend was not significant. In 2020, it declined by nearly 18% and continued to decrease in 2021. Thus, the volume of these services in 2021 accounted only for 75% of the 2019 volume (Fig. 1A).

The most notable decrease between 2019 and 2020 occurred in the number of physician consultations provided as part of night and holiday care in outpatient settings (48%), physician home visits (39%), and nurse outpatient and home visits (34%). However, the quantity of these services had already been gradually decreasing in the pre-pandemic period, though at a smaller pace. The increase in the number of these services observed in 2021 was not able to compensate for the earlier reduction, and utilization in 2021 remained below the values forecasted based on pre-pandemic utilization trends (Table 3).

**Table 3:** Primary health care visits

Types of health services	N (thousands)								*p*-value^&^		% change		
	2015	2016	2017	2018	2019	2020	2021		2015/2016–2019		2020/ 2019	2021/ 2020	2021/ 2019
Outpatient consultations/visits													
physicians	142322.9	148283.8	149135.6	147264.9	150472.0	123529.3	112116.5		NS		-17.9%	-9.2%	-25.5%
nurses	n/d	14308.5	13935.4	13552.7	13398.1	8883.4	10917.1		0.015		-33.7%	22.9%	-18.5%
midwives	n/d	1761.2	1805.0	1702.9	1764.9	1490.9	1706.0		NS		-15.5%	14.4%	-3.3%
night and holiday physician care	5587.4	5726.3	5302.6	5246.5	5135.5	2676.7	3077.2		0.046		-47.9%	15.0	-40.1%
Home visits													
physicians	1816.7	1836.8	1825.9	1685.1	1595.7	981.3	996.1		0.050		-38.5%	1.5	-37.6%
nurses	n/d	7159.5	6852.2	6619.2	6176.6	4088.4	4121.8		0.008		-33.8%	0.8	-33.3%
midwives	n/d	964.1	1060.3	958.0	926.5	738.3	708.6		NS		-20.3%	-4.0%	-23.5%
night and holiday physician care	234.4	222.1	192.8	174.8	155.9	132.1	123.4		0.001		-15.3%	-6.6%	-20.8%

n/d– no data

^&^– *p*-value for linear trend

NS– Not Significant, p>0.05

**Fig. 2:**
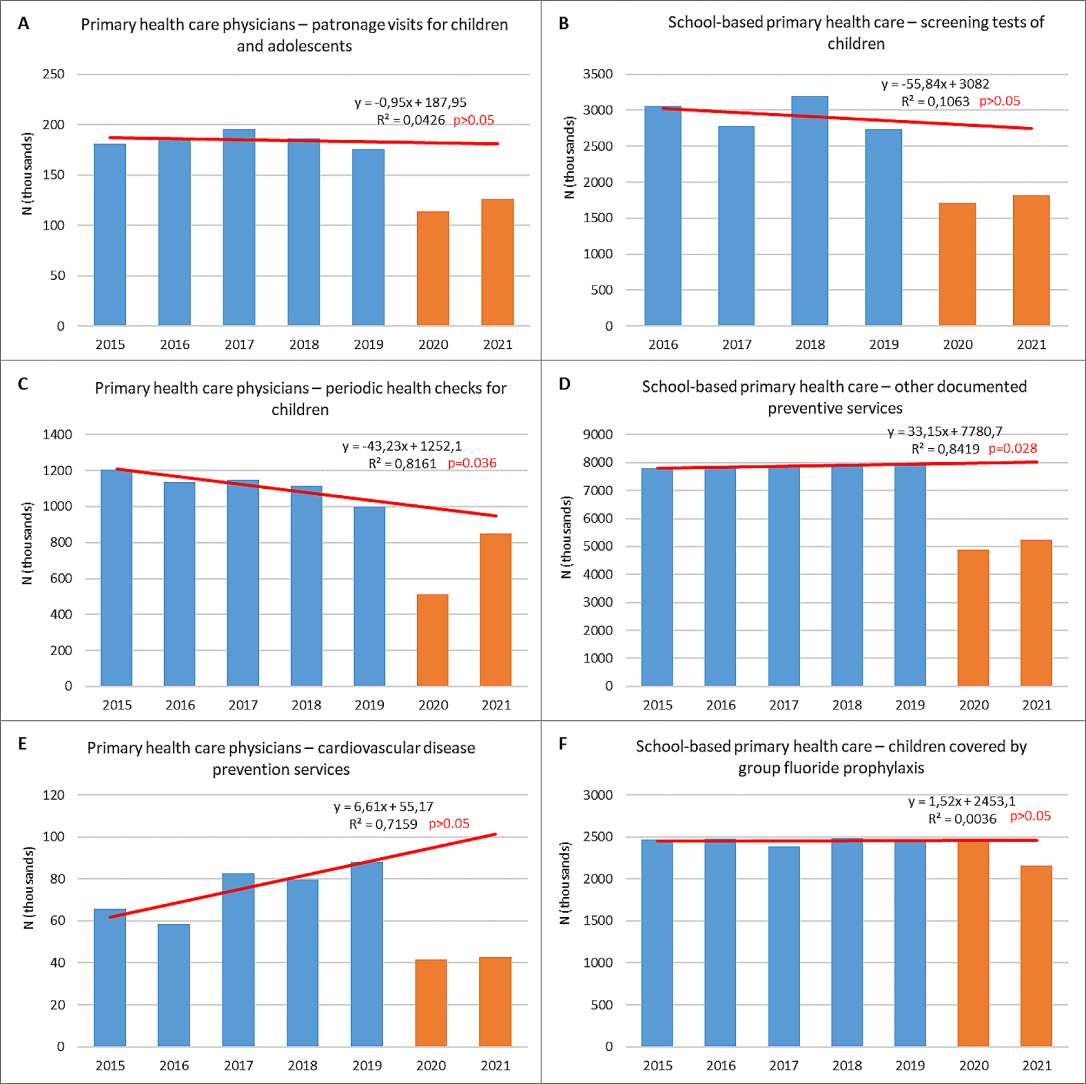
The utilization of selected preventive health care services within primary health care in 2015/2016–2021. Blue bars– pre-pandemic years (2015/2016–2019). Orange bars– pandemic years (2020–2021). Red line– linear trend based on pre-pandemic data

Pre-pandemic data on primary care preventive services most often do not indicate significant linear trends in utilization (*p* > 0.05), with the exemption of periodic health check for children (downward trend), patronage visits for children aged 6 and less (downward trend) and other documented preventive services in schools (upward trend) (Table 4; Fig. 2). Nevertheless, we observed a substantial reduction in their provision between 2019 and 2020. The exemptions are the numbers of midwife patronage visits and children covered by the fluoride prophylaxis, for which, however, there was a decrease in 2021. The drop in the number of services in 2020 was not compensated for in 2021. Thus, the decrease in provision between 2019 and 2021 was still significant, ranging from 10 to 52%, while an increase was observed only in the number of midwives services in the cervical cancer prevention (Appendix 1 Figure C).

**Table 4:** Primary health care preventive services

Types of health services	N (thousands)								*p*-value^&^		% change		
	2015	2016	2017	2018	2019	2020	2021		2015/2016–2019		2020/ 2019	2021/ 2020	2021/ 2019
Primary health care physicians													
Patronage visits for children and adolescents	180.7	186.8	195.3	186.7	176.0	113.6	126.3		NS		-35.5%	11.2	-28.2%
Periodic health checks for children	1206.7	1137.1	1151.1	1116.0	1001.1	514.0	851.5		0.036		-48.7%	65.7%	-14.9%
Cardiovascular disease prevention services	65.8	58.6	82.6	79.7	88.3	41.5	42.7		NS		-53.1%	3.0	-51.7%
Primary health care nurses													
Patronage visits for children aged 6 and less	124.0	127.7	120.7	117.1	107.7	69.9	63.9		0.041		-35.1%	-8.6%	-40.7%
Children who underwent screening tests	n/d	907.8	862.3	879.9	900.1	609.6	706.0		NS		-32.3%	15.8	-21.6%
Primary health care midwives													
Patronage visits	1440.1	1514.9	1609.7	1573.0	1539.8	1887.8	1291.1		NS		22.6	-31.6%	-16.1%
Visits with antenatal education for pregnant women^$^	n/d	1143.4	1195.8	1210.6	1329.3	1085.0	1195.3		NS		-18.4%	10.2%	-10.1%
Services in the cervical cancer prevention	3.8	3.4	3.1	3.1	5.1	4.3	6.6		NS		-14.7%	53.4%	30.8%
School-based primary health care													
Screening tests of children	n/d	3061.4	2777.9	3193.6	2736.7	1708.5	1816.6		NS		-37.6%	6.3	-33.6%
Other screening tests of children^@^	n/d	1981.9	1869.1	1941.9	2153.0	888.0	1133.3		NS		-58.8%	27.6	-47.4%
Other documented preventive services	7791.4	7855.7	7903.9	7929.0	7920.5	4878.5	5239.1		0.028		-38.4%	7.4	-33.9%
Children covered by group fluoride prophylaxis	2468.2	2476.7	2386.7	2484.9	2471.7	2443.6	2161.0		NS		-1.1	-11.6%	-12.6%

n/d– no data

^&^– *p*-value for linear trend

NS– Not Significant, p>0.05

^$^– From 21 weeks of pregnancy

^@^– In other age groups than those specified for screening

#### Outpatient specialist care

Outpatient specialist care, which includes specialist visits and separately financed cost-intensive diagnostic services, was not unaffected by the pandemic (Table 5). The number of consultations with a specialist (conservative and interventional) in 2016–2019 fluctuated slightly, then decreased by approximately 20% in 2020 and remained unchanged in 2021 (Fig. 1B). The quantity of cost-intensive diagnostic services, which had been successively increasing before the pandemic with a statistically significant upward trend in the cases of colonoscopy, computed tomography, and magnetic resonance imaging, also decreased in 2020. The reduction was particularly high for gastroscopy and colonoscopy, i.e. 32% and 24%, respectively. However, the growth in the number of cost-intensive diagnostic services observed in 2021 allowed for the resumption of the 2019 provision and restoring pre-pandemic utilization trends (Appendix 1 Figure D).

**Table 5:** Outpatient specialist visits and cost-intensive diagnostic services

Types of health services	N (thousands)								*p*-value^&^		% change		
	2015	2016	2017	2018	2019	2020	2021		2015/2016–2019		2020/ 2019	2021/ 2020	2021/ 2019
Specialist visits													
Conservative consultation	n/d	76624.8	74507.9	70278.4	72360.4	58128.3	57937.4		NS		-19.7%	-0.3%	-19.9%
Interventional consultation	n/d	8915.2	8811.7	8413.4	9052.2	7049.5	7074.7		NS		-22.1%	0.4	-21.8%
Cost-intensive diagnostic services													
Nuclear medicine diagnostics	96.6	101.4	98.5	101.0	108.0	86.6	106.7		NS		-19.8%	23.1%	-1.3%
Gastroscopy	414.3	427.0	405.5	433.0	458.9	312.5	439.5		NS		-31.9%	40.7%	-4.2%
Colonoscopy	223.7	251.3	305.4	285.0	336.3	255.6	359.4		0.024		-24.0%	40.6%	6.9%
Computed tomography	1118.5	1160.7	1299.8	1265.3	1410.8	1104.4	1532.2		0.018		-21.7%	38.7%	8.6%
Magnetic resonance imaging	743.7	774.8	910.7	996.1	1315.6	1216.3	1664.3		0.017		-7.5%	36.8%	26.5%

n/d– no data

^&^– *p*-value for linear trend

NS– Not Significant, p>0.05

#### Inpatient hospital care

The number of hospitalizations, which was rather stable before the pandemic, decreased by 25% in 2020 compared to 2019, even though the number includes hospitalizations related to COVID-19 (Fig. 1C). Similarly, there was a decrease in the quantity of selected specialist health care services, for which the NHF publishes data, such as arthroplasty and cataract surgery. The volume of these services in the pre-pandemic period 2016–2019 was increasing, with a statistically significant upward trend for primary total knee arthroplasty and cataract surgeries. We can observe only a partial restoration of the previously performed quantity of these procedures in 2021, i.e. up to 92% for hip arthroplasty, however, much below the expected values, taking into account pre-pandemic trends (Table 6).

**Table 6:** Hospital services

Types of health services	N (thousands)								*p*-value^&^		% change		
	2015	2016	2017	2018	2019	2020	2021		2015/2016–2019		2020/ 2019	2021/ 2020	2021/ 2019
Inpatient stays	10498.3	10102.3	10235.3	10261.0	10133.2	7617.9	8088.8		NS		-24.8%	6.2	-20.2%
Arthroplasty of the joint^$^	n/d	71.0	85.4	88.1	92.8	69.5	84.2		NS		-25.1%	21.1%	-9.3%
Primary total hip arthroplasty	n/d	36.7	43.6	44.5	47.0	34.9	43.1		NS		-25.7%	23.3%	-8.3%
Primary total knee arthroplasty	n/d	18.9	24.7	26.7	29.3	20.7	25.3		0.031		-29.3%	22.2%	-13.6%
Cataract surgeries	n/d	245.4	301.1	309.9	355.6	233.7	297.6		0.031		-34.3%	27.3%	-16.3%

n/d– no data

^&^– *p*-value for linear trend

NS– Not Significant, p>0.05

^$^– hip, knee, elbow, shoulder or another / primary and revision / total and partial

#### Preventive health programmes

The implementation of preventive programmes financed by the NHF outside of primary health care was significantly limited during the COVID-19 pandemic compared to 2015–2019. The volume of services provided under the tobacco-related disease prevention programme decreased by nearly 70% between 2019 and 2020, and by nearly 32% in the next year. The number of services provided under the cervical cancer prevention programme was already decreasing before the pandemic, but the reduction observed in 2020, which was largely maintained into 2021, was much greater than pre-pandemic trend. The use of breast cancer prevention programme services decreased in 2020, but then their provision was nearly fully resumed in 2021 (Table 7, Appendix 1 Figure F).

**Table 7:** Preventive health programmes

Types of health services #	N (thousands)								*p*-value^&^		% change		
	2015	2016	2017	2018	2019	2020	2021		2015–2019		2020/ 2019	2021/ 2020	2021/ 2019
Tobacco-related disease prevention programme	6.8	6.2	6.8	7.0	5.1	1.6	1.1		NS		-69.4%	-31.6%	-79.0%
Cervical cancer prevention programme	595.1	555.3	504.4	505.4	471.2	282.3	354.4		0.008		-40.1%	25.6%	-24.8%
Breast cancer prevention programme	1024.3	1004.1	1012.1	1005.7	1024.3	722.9	976.6		NS		-29.4%	35.1%	-4.7%

#–number of services provided in the basic stage of the programmes

^&^– *p*-value for linear trend

NS– Not Significant, p>0.05

#### Psychiatric care and addiction treatment

The number of inpatient stays within psychiatric care and addiction treatment decreased between 2019 and 2020 by 24% (Table 8). Such a large decline was not expected even taking into account the significant downward trend before the pandemic. In 2021, the number of stays increased to almost match the pre-pandemic utilization trend. At the same time, the number of patients covered by stationary psychiatric care was increasing during the two pandemic years. However, just before the pandemic, in 2019, this number decreased by 10%, and the increase in 2020 was close to covering this reduction (Fig. 1D). The use of psychiatric day care, stable before the pandemic, was significantly lower in 2020 and still was in 2021 compared 2019. The pandemic seems to have little or no negative impact on the use of outpatient psychiatric care.

The number of patients in addiction treatment was relatively stable during the pre-pandemic period. In 2020, the utilization decreased across all settings, but particularly in stationary care. The drop in 2020 was higher than the increase observed in the following year.

**Table 8:** Psychiatric care and addiction treatment services

Types of health services	N (thousands)								*p*-value^&^		% change		
	2015	2016	2017	2018	2019	2020	2021		2015/2016–2019		2020/ 2019	2021/ 2020	2021/ 2019
Inpatient stays (psychiatric care and addiction treatment)	286.8	280.7	280.4	262.8	252.3	192.7	225.1		0.013		-23.6%	16.8%	-10.8%
Psychiatric care													
Number of patients in stationary care	n/d	153.7	154.8	152.8	137.5	150.8	185.1		NS		9.7%	22.8%	34.6%
Number of patients in outpatient care	n/d	1350.2	1360.8	1332.9	1248.5	1202.4	1302.3		NS		-3.7%	8.3%	4.3%
Number of patients in day care	n/d	27.6	27.7	27.9	27.9	21.4	24.4		NS		-23.1%	13.7%	-12.5%
Addiction treatment													
Number of patients in stationary care	n/d	75.9	76.8	72.4	72.8	56.3	61.4		NS		-22.6%	9.0	-15.6%
Number of patients in outpatient care	n/d	235.3	233.6	229.3	232.4	203.1	216.9		NS		-12.6%	6.8%	-6.7%
Number of patients in day care	n/d	11.3	11.3	11.1	11.1	8.1	10.0		NS		-26.7%	22.7%	-10.0%

n/d– no data

^&^– *p*-value for linear trend

NS– Not Significant, p>0.05

#### Medical rehabilitation and health resort rehabilitation

There was significant decrease in the use of medical rehabilitation during the first year of the pandemic, across different settings including the most frequently used services, i.e. outpatient physiotherapy treatment (Fig. 1E). Only the number of patients in home physiotherapy treatment continued to grow during the pandemic period following the pre-pandemic trend. In 2020, health resort rehabilitation also decreased, even more significantly than for medical rehabilitation. The increase in the provision of rehabilitation in 2021 was small compared to the earlier drop, making this area of health care one of the most affected by the pandemic (Table 9).

**Table 9:** Medical and health resort rehabilitation services

Types of health services	N (thousands)								*p*-value^&^		% change		
	2015	2016	2017	2018	2019	2020	2021		2015–2019		2020/ 2019	2021/ 2020	2021/ 2019
Medical rehabilitation													
Inpatient stays	251.5	240.4	233.8	234.5	237.6	159.6	176.3		NS		-32.8%	10.5	-25.8%
Number of patients in stationary care	234.1	227.2	220.9	223.2	209.4	151.5	165.4		0.022		-27.7%	9.2	-21.0%
Number of patients– outpatient physiotherapy treatment	2654.2	2732.2	2766.2	2568.7	2464.6	1868.9	2064.3		NS		-24.2%	10.5	-16.2%
Number of patients– home physiotherapy treatment	8.5	36.6	37.8	45.0	75.6	87.7	118.4		0.017		16.0%	35.0%	56.6%
Number of patients– centre/day ward	264.3	282.5	286.7	308.3	344.0	280.3	317.5		0.010		-32.8%	10.5	-25.8%
Health resort rehabilitation													
Inpatient stays	53.0	59.8	57.5	54.9	51.3	23.7	27.3		NS		-53.8%	15.4	-46.7%
Number of patients in stationary care (sanatorium or spa hospital)	388.5	391.7	385.9	388.9	391.5	198.9	259.7		NS		-49.2%	30.6	-33.7%
Number of patients in outpatient care	16.0	16.2	15.8	15.2	14.6	5.4	5.9		0.028		-63.1%	9.7	-59.5%

^&^– *p*-value for linear trend

NS– Not Significant, p>0.05

#### Long-term care and palliative care

In long-term care, there was no significant reduction in the number of patients and stays in stationary care in 2020 compared to previous years. The number of patients using the most frequently provided services, i.e. home long-term care nurse services, remained rather stable during the pandemic (Fig. 1F). However, the number of people requiring home care provided by a team for mechanically ventilated patients increased in the two analysed years of the pandemic, following the pre-pandemic trend, which may, however, be due to COVID-19 patients. On the other hand, the use of palliative care (in all settings) was reduced in 2020 and increased only slightly in 2021 for all types of palliative care but outpatient care. The number of patients in outpatient care continued to decrease in 2021, though it should be noted that the utilization of these services also fluctuated in the pre-pandemic years (Table 10).

**Table 10:** Long-term care and palliative care services

Types of health services	N (thousands)								*p*-value^&^		% change		
	2015	2016	2017	2018	2019	2020	2021		2015/2016–2019		2020/ 2019	2021/ 2020	2021/ 2019
Long-term care													
Inpatient stays	n/d	29.3	36.3	33.5	34.7	33.5	34.6		NS		-3.6%	3.4%	-0.3%
Number of patients in stationary care	40.7	40.7	43.7	42.7	41.9	40.9	44.6		NS		-2.4%	9.0%	6.4%
Number of patients in home care (team for mechanically ventilated patients)	4.4	4.9	7.2	6.8	7.7	8.2	8.9		0.027		6.5%	8.8%	15.9%
Number of patients in home care (long-term care nurse)	63.7	60.1	65.2	62.0	60.8	59.4	60.6		NS		-2.4%	2.0%	-0.4%
Palliative care													
Inpatient stays	n/d	36.3	38.1	40.7	39.3	32.5	34.1		NS		-17.3%	5.0	-13.2%
Number of patients in stationary care	33.2	33.0	33.4	35.8	36.3	30.2	31.9		0.036		-16.9%	5.8	-12.0%
Number of patients in outpatient care	14.2	13.0	18.1	14.1	13.9	11.2	10.0		NS		-19.2%	-10.5%	-27.7%
Number of patients in home care	56.9	56.1	55.0	61.6	62.7	61.9	63.7		NS		-1.3%	2.9%	1.6%

n/d– no data

^&^– *p*-value for linear trend

NS– Not Significant, p>0.05

## Discussion

The aim of this study was to assess the impact of the COVID-19 pandemic on the provision and utilization of public health care services in Poland. With qualitative data, we have shown how the principles of health care provision were altered due to the pandemic, and using quantitative data we have presented the changes in the number of health care users and the volume of provided services.

### Summary of results and their explanation

The results of our analysis showed that, although public authorities in Poland took certain actions to ensure the availability of public health care services during the pandemic (e.g. e-visits, additional funds for providers to cover the cost of the elevated sanitary regime), there was a reduction in the use of services in the first year of the pandemic (2020) across all scopes of health care, which in some cases disrupted statistically significant pre-pandemic trends in health care utilization. At the same time, policy was implemented to pay providers for contracted services regardless of their activity (advance payments), to secure the providers’ income when demand was low, and provision of services was hindered. In 2021, the utilization of most services increased as providers and patients adjusted to the pandemic conditions. Providers who had earlier received advanced payments were also obliged to make up for the lower provision of health care services in 2020, and the reimbursement limits of some services were lifted. However, most often the increase was not sufficient to compensate for the earlier drop, thus, after the two studied years of the pandemic, the utilization was below pre-pandemic levels. Also, other measures implemented during the pandemic, such as e-visits, though most likely improving access to care during the pandemic, did not encourage the resumption of traditional visits, which were necessary in some cases. This problem was acknowledged by the NHF, and measures had to be implemented to incentivise the provision of face-to-face visits.

We observed significant differences in the impact of the pandemic on health care use across various health care services. The smallest fluctuations in the number of services provided were observed in the case of long-term care. This is due to the specificity of this area of health care, with its focus on care and nursing services, and with nearly complete permanent occupancy of the available beds and long waiting lists [59]. Long-term care facilities were not closed to patients during the COVID-19 pandemic. There were also specific services, the use of which continued to grow during the pandemic reaching levels only slightly below the expected values based on pre-pandemic upward utilization trends, i.e., drug programmes, home-based physiotherapy treatment, and home-based long-term care for mechanically ventilated patients. All these services are provided for patients with serious health issues, and forgoing their use would have involved a high risk for health and life. Our results also show that in some areas of health care the provision of services was resumed faster than in others. These are for example cost-intensive diagnostic services (magnetic resonance imaging, computed tomography, and colonoscopy), which are provided within outpatient specialist care and are paid for separately using the fee-for-service method. The use of these diagnostic services decreased in 2020 but then increased significantly in the second year of the pandemic following pre-pandemic utilization trends.

The largest reduction in the number of provided health services during the two years of the COVID-19 pandemic occurred in rehabilitation, especially health resort rehabilitation, as well as preventive services, particularly periodic health checks and screening tests for children, cardiovascular disease prevention (all provided within primary care), and the tobacco-related disease prevention programme. Although for the majority of these services we did not observe statistically significant utilization trends based on pre-pandemic data, the utilization during the pandemic years fell well below the level from any pre-pandemic year. Therefore, we can assume some impact of the pandemic on the use of these services. For rehabilitation services, the decrease in utilization might result from the temporary closure of rehabilitation facilities and the inability to provide these services as teleconsultations, while providers still could receive payments for contracted services. Due to the closure of schools, certain preventive services for children could not be provided either. Primary care providers also did not have incentives to provide care as selected preventive services were paid for using a non-activity-based method, i.e., capitation. The evidence available in the literature indicates that the capitation method led to an increase in profitability for primary care providers during the COVID-19 pandemic due to the reduction of provided services and cost savings [35].

Both areas of health care largely affected by the pandemic (rehabilitation and prevention) are of a non-curative nature, while the health outcomes of these services are often delayed in time. Thus, they are usually undervalued by both decision makers and patients themselves [75]. However, the lack of access or delayed implementation of preventive health programmes and screening tests may lead to late disease diagnosis, which significantly worsens the prognosis. The effects of the lack of preventive health services in children may be equally detrimental and visible only after many years [76]. Based on the countries’ experiences with COVID-19, one of the defined priorities to enhance health system resilience is to strengthen the role of public health interventions [77]. Similarly, in the absence of or delayed rehabilitation, there is a risk of a significant deterioration in health and capacity for work. This may increase disability-related expenditure such as pensions and care benefits [78].

### Comparison with the literature

Our results are in line with the findings of other studies reporting on pandemic-related changes in overall health care utilization. In the systematic review by Moynihan R. et al. [1], which covered 81 studies across 20 countries and evaluated the impact of the pandemic on several areas of health care until May 2020, the percentage change in the use of health services ranged from a 49% increase to an 87% decrease with a median of– 37.2% (IQR − 51% to − 20%). Some increases have been observed, e.g. in telemedicine, but most of the reviewed studies have indicated a reduction in health care use. In our analysis, the decrease in the number of provided services in 2020 compared to 2019 ranged from a 3.6% (for long-term care) to a 69.4% (for the tobacco-related disease prevention programme), and only few increases in the number of services were observed, i.e. up to 22.6% increase for midwife patronage visits.

Previous Polish studies on specific scopes of services and/or groups of patients point to similar direction of changes in health care utilization due to the COVID-19 pandemic as our study:

- Primary health care: The studies assessing primary health care in the period March-November 2020 [35] and April-July 2020 [36] showed, that the number of services provided during the pandemic was lower than before the pandemic (up to 100% decrease for home visits). These studies did not analyse all primary health care services e.g. screening tests in children, which was one of most affected by the pandemic as our analysis showed.
- Preventive health programmes: The study by Poniewierza et al. 2022 showed a negative impact of the COVID-19 pandemic on the number of patients in a cervical cancer screening programme in the public health care system in 2020–2021 [30]. Our results indicated a significant decrease in the number of provided services within the programme in 2020, and then some increase in 2021. Other studies on the universal newborn hearing screening programme [31] and the prenatal screening programme [32] indicated a reduction in the number of services provided in 2020 [31, 32] and the restoration of the programme in 2021 in most of the analysed facilities [31]. In our study we did not analyse these specific programmes, but nearly full resumption of pre-pandemic utilization occurred for only one out of the three programmes that we analysed (the breast cancer prevention programme).
- Dental care: The study on dental care [27] showed that in April 2020 compared to April 2019, the number of services decreased by more than 23 times [27]. In another study by Nijakowski et al. 2021, it was indicated that in 2020 the number of conservative procedures was particularly reduced [40]. In our study, based on annual country-level data, we showed that the number of patients using dental care decreased in 2020 compared to 2019 by 23%.
- Hospital care and outpatient specialist care: In our study we observed a significant reduction in the number of specialist visits and inpatient stays in 2020, which was not made up for in 2021. Studies on selected services within hospital or specialist care also demonstrated the negative impact of the COVID-19 pandemic on the provision of health services, including cancer diagnosis and treatment [28, 29], diabetes treatment [37], surgical heart disease treatment or cardiohematology [39, 47], urologic emergency visits and admissions [44], hospitalizations at dermatology department [45], sexually transmitted infections treatment [46] and arthroplasty [41, 42]. Only a single-centre study by Kazubski et al. 2021 [43] found a neutral impact of the COVID-19 pandemic on hospitalizations related to knee or shoulder arthroscopy in the period from March 4 to October 15, 2020 compared to the same period in 2019.
- In our work, we did not evaluate the number of transplants because such data is not published by the NHF. However, the study based on data published by Poltransplant indicated that the number of solid organ transplants decreased in Poland by over 35% in 2020 [34].
- Results of surveys conducted among Polish patients or medical staff also confirm the conclusions drawn from our study. Patients had significantly fewer visits to their general practitioner [38], use less preventive services [53], as well as reported barriers to access health services [54] and their treatment was postponed [51]. The access to health care was limited during the pandemic also in the opinion of medical staff (e.g. urological consultation [48], bariatric care [49]), and the pandemic had a significantly negative impact on the activities of medical facilities and staff [50, 52].

### Limitations

Our analysis is the first conducted specifically for the Polish setting that includes country-level data for all types of publicly financed health care, which makes it possible to compare changes in utilization during the two years of the COVID-19 pandemic across different scopes of health services. Nevertheless, our study has several limitations.

Based on our analysis we cannot draw conclusions on a causal relation between the pandemic and the observed changes in health care utilization. It is likely that other factors not related to the pandemic might have also influenced the provision of specific services. Health care utilization is determined by the availability of health services, but also, among others, the need for these service (i.e., levels of illness or disability), and the resources available for providing and paying for health service.

Due to the lack of comparable data, we were also unable to include more than five pre-pandemic years and conduct a more robust trend analysis to see how utilization in 2020–2021 deviated from expected levels. The analysis also does not consider the period after 2021 (2022–2023).

Since we did not have data specifically on non-COVID-19 services, our analysis also covers health services provided to patients with COVID-19. Thus, the numbers on health care utilization during the analysed two years of the pandemic could be even lower if one excludes COVID-19 services. Additionally, the analysis covers only health care services within the statutory health care system. We do not include services paid for privately by patients. However, out-of-pocket household health expenditure between 2019 and 2020 remained nearly the same (PLN 29,701.9 million in 2019 compared to PLN 29,668.4 million in 2020 [79], and taking inflation into account, the real household expenditure even decreased in the first year of the pandemic. This indicates that patients did not move to the private sector during the pandemic, and the “health debt” resulting from the reduction in the number of services provided in the public sector has not been reduced by increased use in the private sector.

### Implication of the study

Our results point to the need for Poland to implement policies targeting the areas of care significantly affected by the pandemic (prevention and rehabilitation) to lessen the health consequences of the reduced access during the pandemic.

To improve the provision of preventive and rehabilitation services, cost-based pricing should be used. Particularly, it might be necessary to increase public spending on rehabilitation as these services are underpriced [80], and to withdraw the existing volume limits of rehabilitative services to which patients are entitled. Currently, the provision of these services is unprofitable, and many providers resign from providing them within the public sector, while waiting times for rehabilitative services are very long [80]. The implementation of preventive services could be enhanced though financial incentives, e.g. making the payment of a capitation fee conditional on the achievement of a certain level of service delivery [81]. Financial and non-financial mechanisms can also help change health care consumer behaviour and increase public awareness of the importance of primary and secondary prevention [82].

Our results indicate that during shocks, such as the pandemic, there is a need for mechanisms to quickly adjust to new conditions and challenges in health care provision, taking into account the specificity of different types of health care services, e.g. mode of provision (inpatient/outpatient/home-based), provider payment methods (activity-based/non-activity-based), and needs and demand for specific services. When implementing these measures, decision makers need to balance the interests of various stakeholders, i.e. the financial security of health care providers, access to care for both COVID-19 patients and non-COVID-19 patients. There is also a need to constantly monitor implanted measures to evaluate their effectiveness and mitigate unintended consequences. For example, in Poland, the implementation of e-visits resulted in to reduced access to traditional consultations when conditions already allowed for face-to-face contact.

### Recommendations for further studies

Monitoring the level of health care utilization in the following years should be continued, especially in the areas where the greatest negative impact of the pandemic was observed. Some population groups might have been disproportionally affected by the pandemic. Therefore, it may be important to examine the use of health care by different population groups taking into account social, economic and geographical factors, in order to assess inequalities in access to services and develop strategies to address them.

During and after the pandemic, various mechanisms were introduced to improve the use of and access to health care. These solutions should be evaluated to ensure that the most effective and cost-effective mechanisms are in place to respond to any future pandemics and shocks.

## Conclusions

The COVID-19 pandemic had a significant impact on the number of health services provided in Poland. The reduction in the use of publicly financed health care services, which disturbed pre-pandemic utilization trends, was particularly evident in the first year of the pandemic (2020). Implemented policies were rarely sufficient in restoring the pre-pandemic utilization of health care services during the second year of the pandemic. Our analysis has shown that the areas experiencing the most significant declines of utilization were rehabilitation and prevention. As these services are of particular relevance for health capital of the population, there is a need for additional investments and actions that improve access to and utilization of these services.

### Electronic supplementary material

Below is the link to the electronic supplementary material.

Supplementary Material 1

## Data Availability

The datasets analysed during the current study are available in the NHF repository [https://baw.nfz.gov.pl/NFZ] [57].

## Abbreviations

DRG: Diagnosis-Related Group
NHF: National Health Fund
WHO: World Health Organization
