# Supplementary material for: How COVID-19 has changed the utilization of different health care services in Poland
Source: BMC Health Serv Res. 2024 Jan 18;24:105. doi: 10.1186/s12913-024-10554-7 (PMC10797947; doi:10.1186/s12913-024-10554-7)
Supplement: Supplementary file 1 — Supplementary Material 1 [file 12913_2024_10554_MOESM1_ESM.docx]

**Appendix 1.**

**Figure A.** Health care users in 2015/2016-2021.


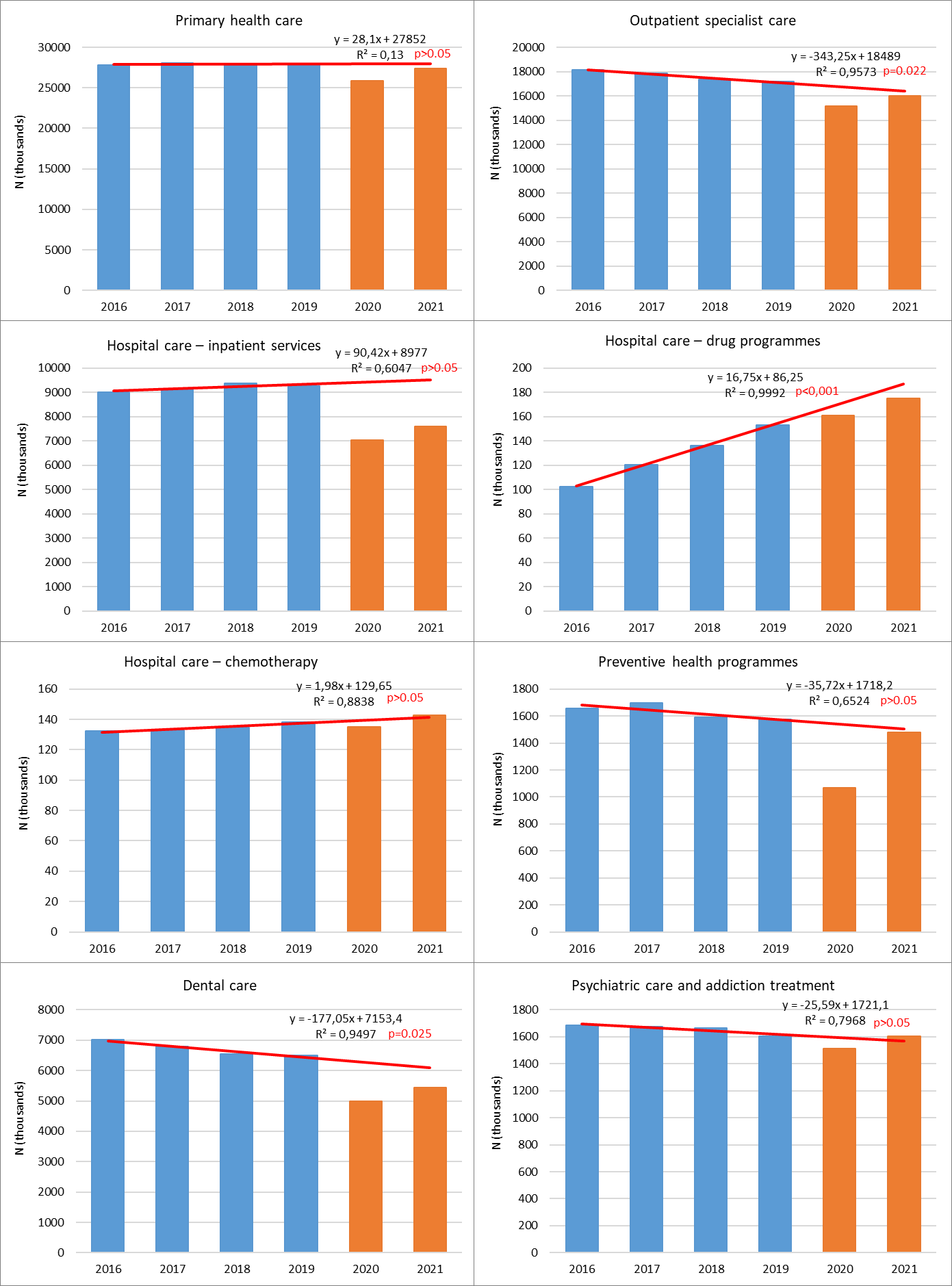


Blue bars – pre-pandemic years (2015/2016-2019); Orange bars – pandemic years (2020-2021); Red line – linear trend based on pre-pandemic data

**Figure A (cont.).** Health care users in 2015/2016-2021.


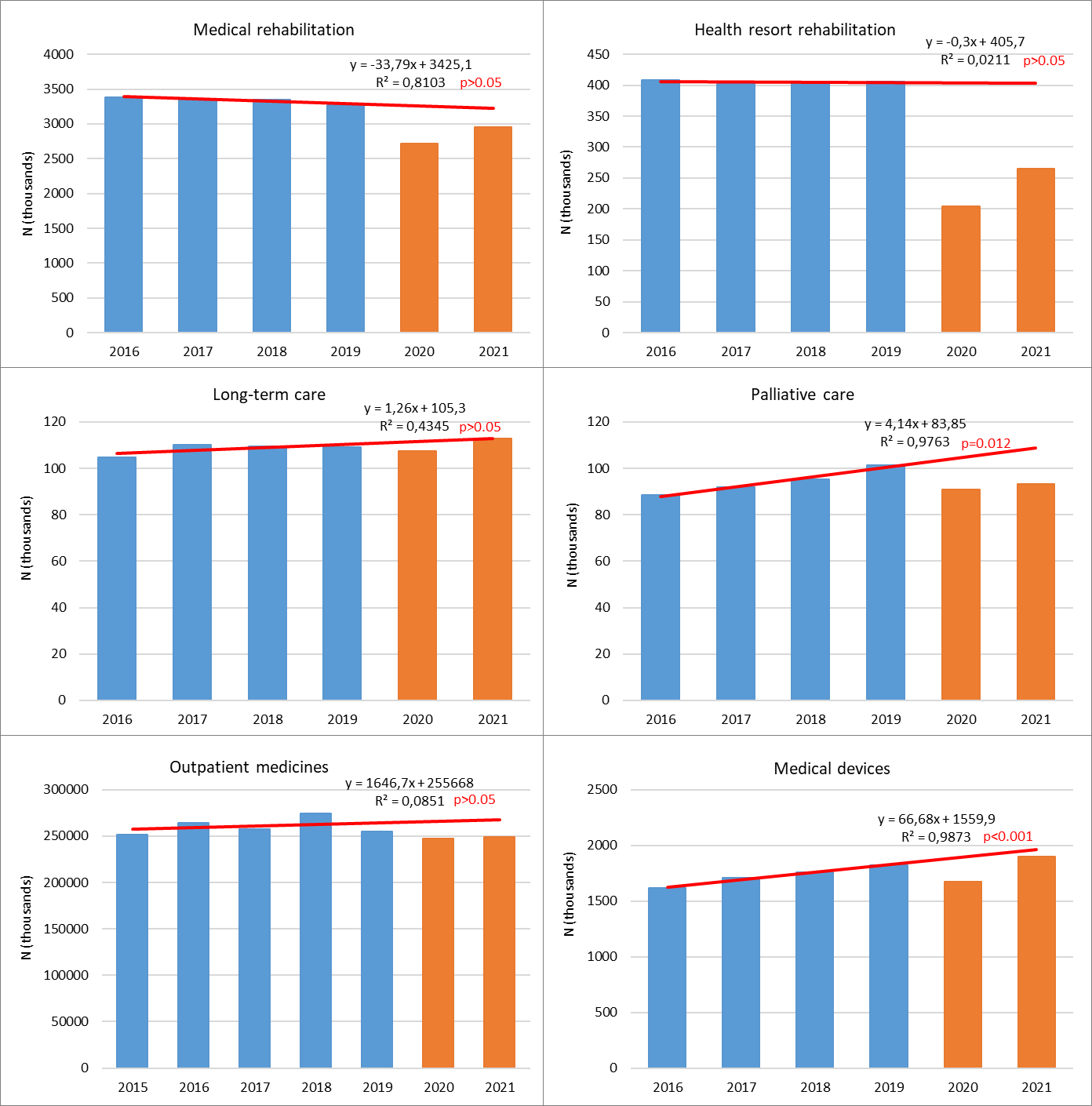


Blue bars – pre-pandemic years (2015/2016-2019); Orange bars – pandemic years (2020-2021); Red line – linear trend based on pre-pandemic data

**Figure B.** The utilization of primary care services in 2015/2016-2021.


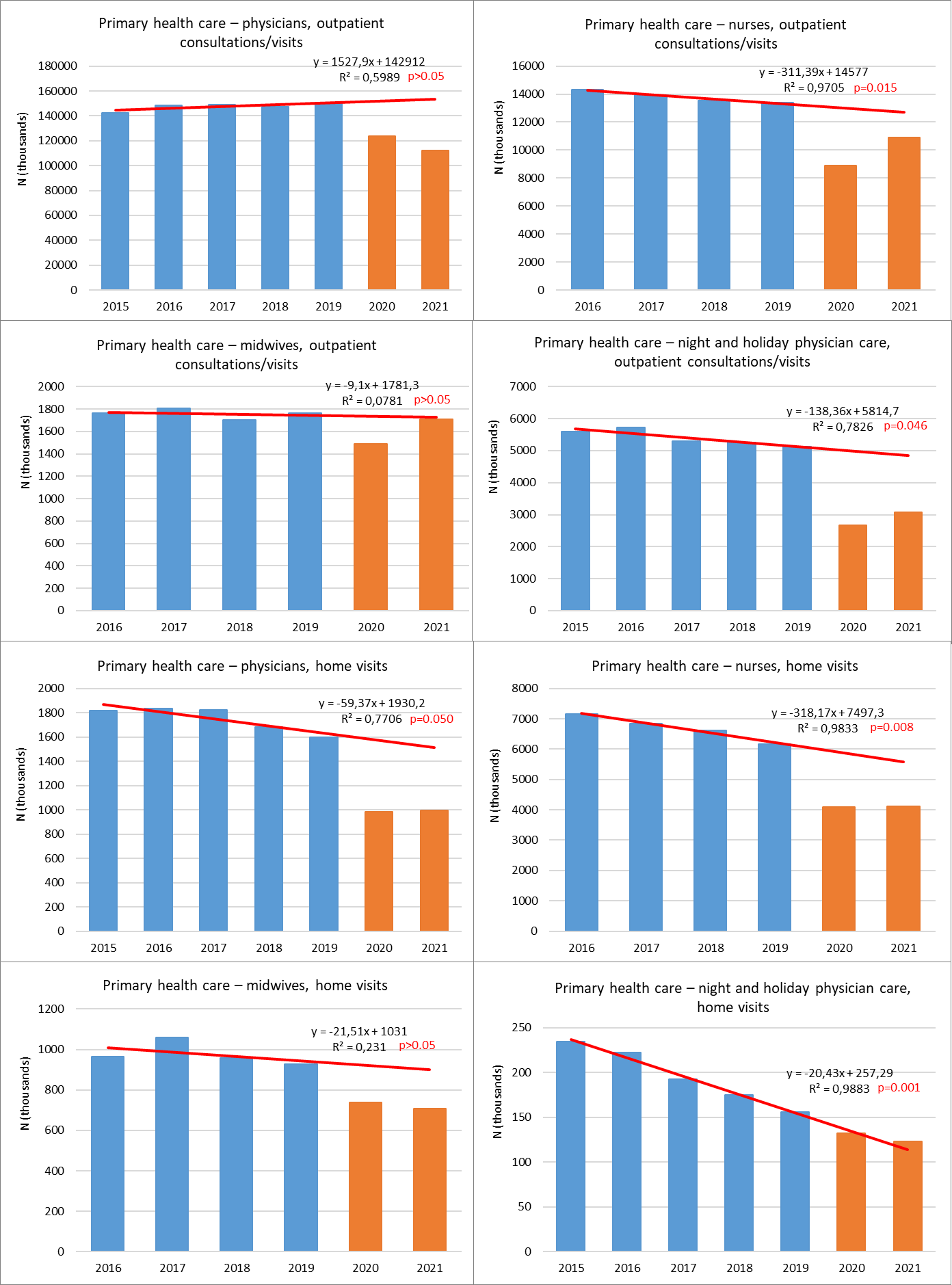


Blue bars – pre-pandemic years (2015/2016-2019); Orange bars – pandemic years (2020-2021); Red line – linear trend based on pre-pandemic data

**Figure C.** The utilization of selected primary health care preventive services in 2015/2016-2021.


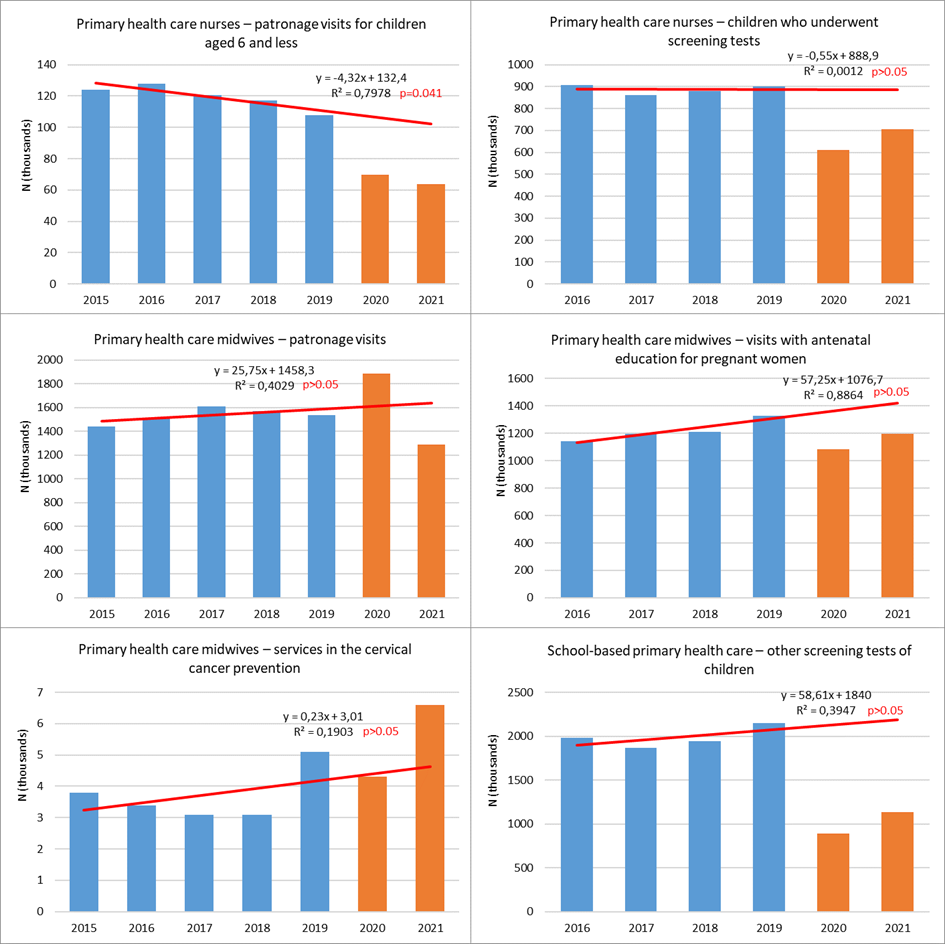


Blue bars – pre-pandemic years (2015/2016-2019); Orange bars – pandemic years (2020-2021); Red line – linear trend based on pre-pandemic data

**Figure D.** The utilization of selected outpatient specialist services and cost-intensive diagnostic services in 2015/2016-2021.


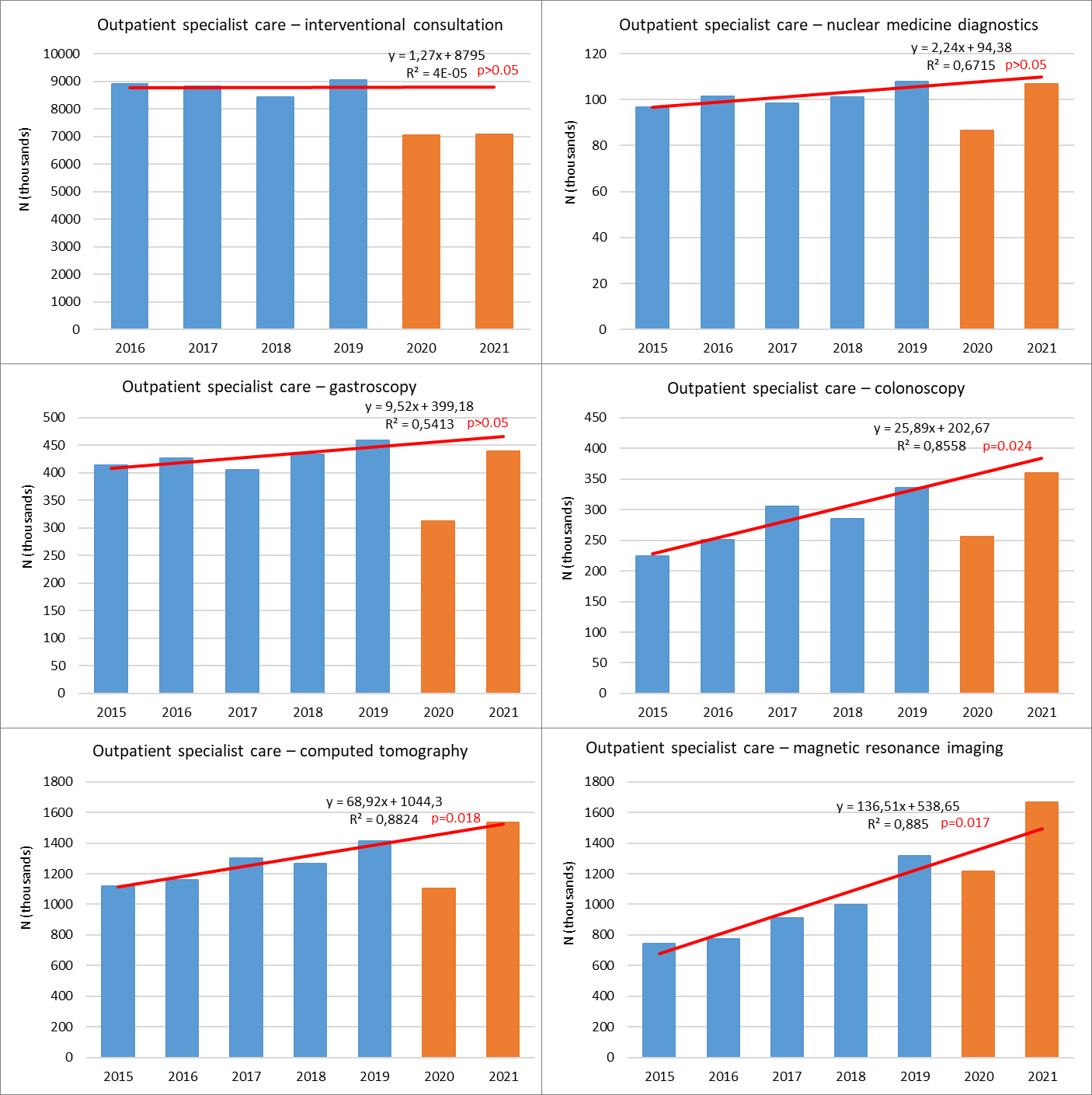


Blue bars – pre-pandemic years (2015/2016-2019); Orange bars – pandemic years (2020-2021); Red line – linear trend based on pre-pandemic data

**Figure E.** The utilization of selected hospital services in 2016-2021.


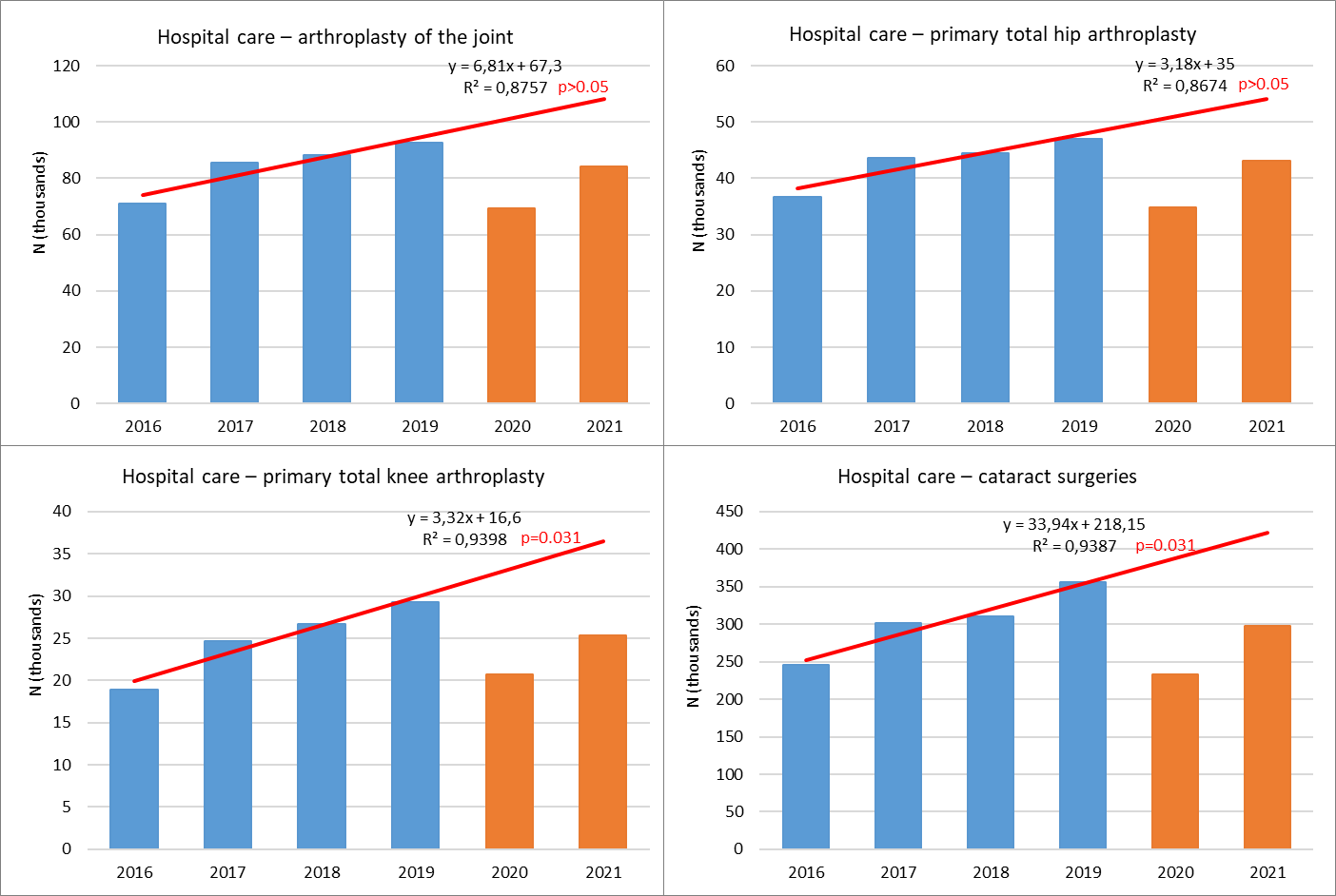


Blue bars – pre-pandemic years (2015/2016-2019); Orange bars – pandemic years (2020-2021); Red line – linear trend based on pre-pandemic data

**Figure F.** The utilization of preventive health programmes services in 2015-2021.


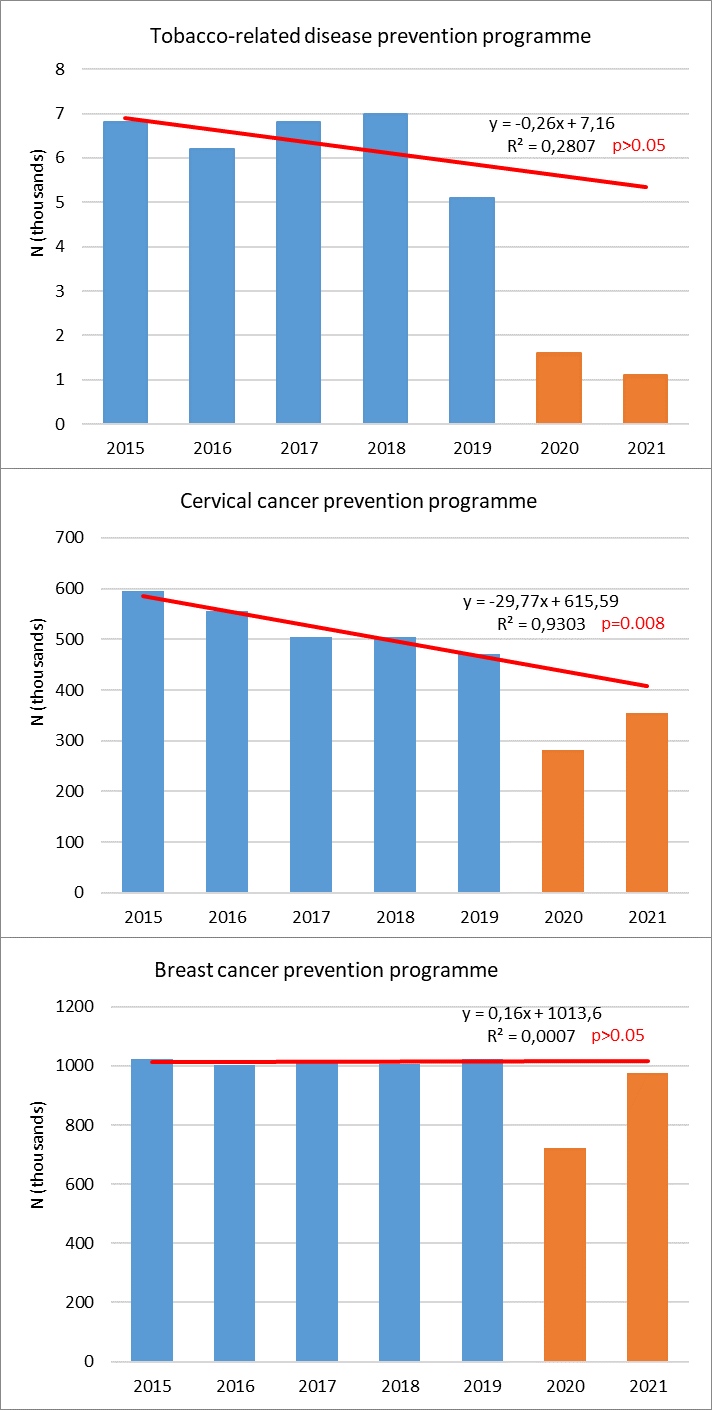


Blue bars – pre-pandemic years (2015/2016-2019); Orange bars – pandemic years (2020-2021); Red line – linear trend based on pre-pandemic data

**Figure G.** The utilization of selected psychiatric care and addiction treatment services in 2015/2016-2021.


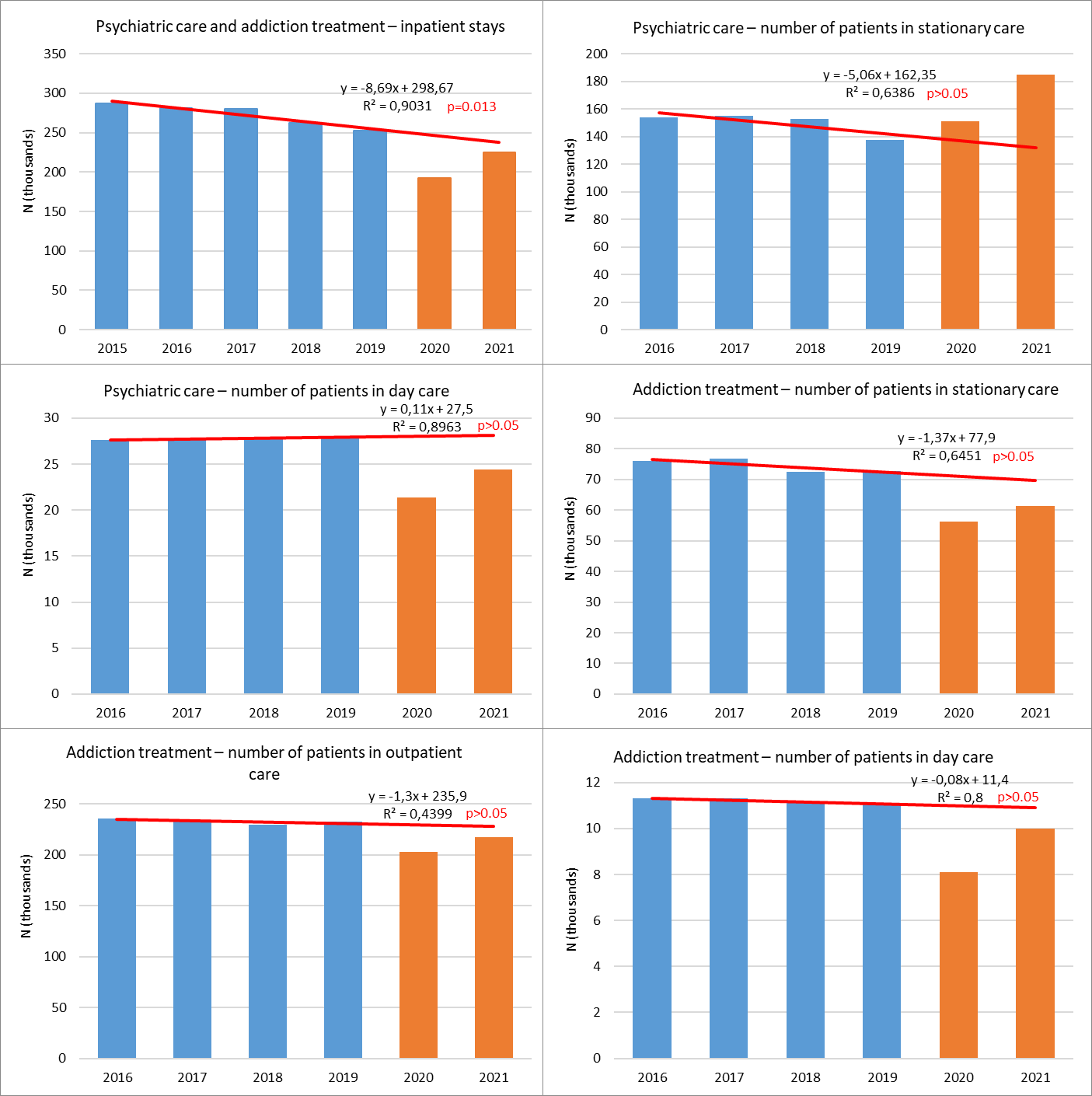


Blue bars – pre-pandemic years (2015/2016-2019); Orange bars – pandemic years (2020-2021); Red line – linear trend based on pre-pandemic data

**Figure H.** The utilization of medical and health resort rehabilitation services in 2015-2021.


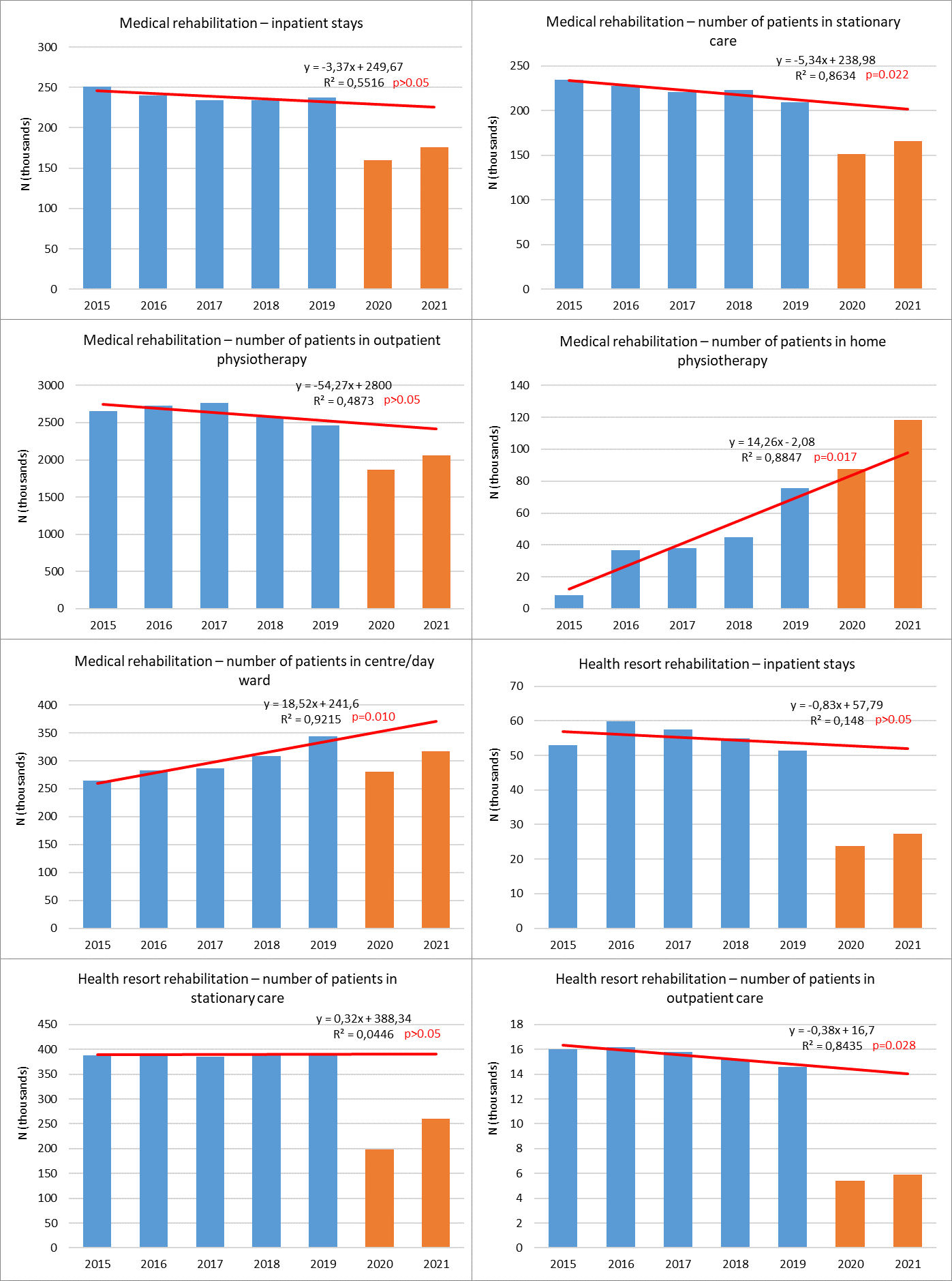


Blue bars – pre-pandemic years (2015/2016-2019); Orange bars – pandemic years (2020-2021); Red line – linear trend based on pre-pandemic data

**Figure I.** The utilization of long-term care and palliative care services in 2015/2016-2021.


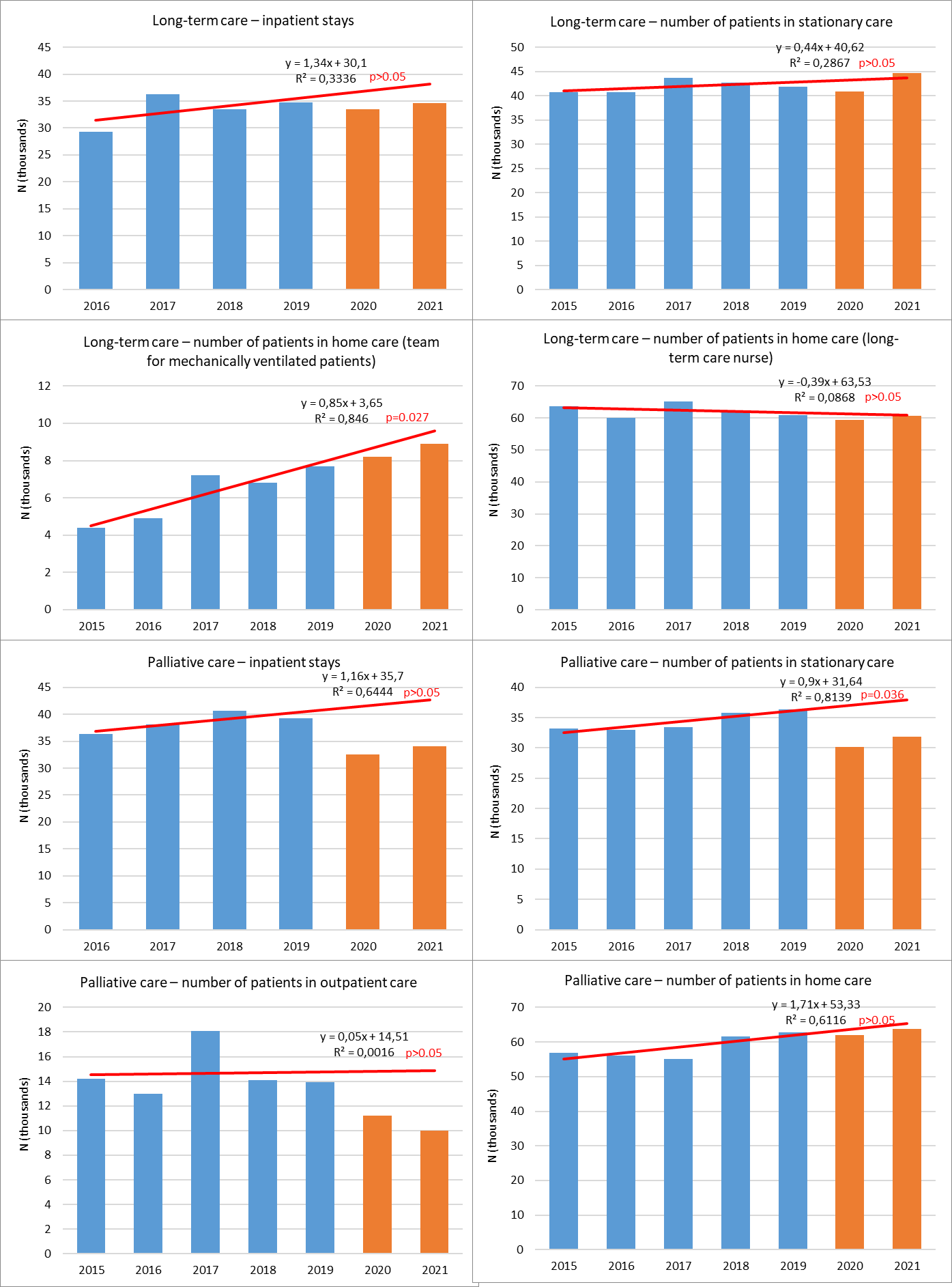


Blue bars – pre-pandemic years (2015/2016-2019); Orange bars – pandemic years (2020-2021); Red line – linear trend based on pre-pandemic data
